# Comparative studies of mitochondrial reactive oxygen species in animal longevity: Technical pitfalls and possibilities

**DOI:** 10.1111/acel.13009

**Published:** 2019-07-19

**Authors:** Daniel Munro, Matthew E. Pamenter

**Affiliations:** ^1^ Department of Biology University of Ottawa Ottawa Ontario Canada; ^2^ University of Ottawa Brain and Mind Research Institute Ottawa Ontario Canada

**Keywords:** antioxidants, *Heterocephalus glaber*, horseradish peroxidase, mitochondria, oxidative stress theory of aging, reactive oxygen species

## Abstract

The mitochondrial oxidative theory of aging has been repeatedly investigated over the past 30 years by comparing the efflux of hydrogen peroxide (H_2_O_2_) from isolated mitochondria of long‐ and short‐lived species using horseradish peroxidase‐based assays. However, a clear consensus regarding the relationship between H_2_O_2_ production rates and longevity has not emerged. Concomitantly, novel insights into the mechanisms of reactive oxygen species (ROS) handling by mitochondria themselves should have raised concerns about the validity of this experimental approach. Here, we review pitfalls of the horseradish peroxidase/amplex red detection system for the measurement of mitochondrial ROS formation rates, with an emphasis on longevity studies. Importantly, antioxidant systems in the mitochondrial matrix are often capable of scavenging H_2_O_2_ faster than mitochondria produce it. As a consequence, as much as 84% of the H_2_O_2_ produced by mitochondria may be consumed before it diffuses into the reaction medium, where it can be detected by the horseradish peroxidase/amplex red system, this proportion is likely not consistent across species. Furthermore, previous studies often used substrates that elicit H_2_O_2_ formation at a much higher rate than in physiological conditions and at sites of secondary importance in vivo. Recent evidence suggests that the activity of matrix antioxidants may correlate with longevity instead of the rate of H_2_O_2_ formation. We conclude that past studies have been methodologically insufficient to address the putative relationship between longevity and mitochondrial ROS. Thus, novel methodological approaches are required that more accurately encompass mitochondrial ROS metabolism.

## 1 INTRODUCTION

Life‐extending treatments, such as caloric restriction, typically increase murine lifespan/healthspan by 30%–35% (Masoro, 2006), whereas methionine restriction achieves a 10%–20% increase (Scott McIsaac, Lewis, Gibney, & Buffenstein, 2016). In contrast, natural variations in lifespan between similarly sized species within the same order can be as great as 7.5‐fold (e.g., rodentia; mice vs. naked mole‐rats, NMRs), and as great as 30‐fold across placental mammals (e.g., pigmy shrews vs. little brown bats). The comparative approach therefore holds great promise for the elucidation of novel insights into the physiological mechanisms that modulate animal lifespan and healthspan, which are often used in orienting translational therapeutic avenues.

The physiological causes of aging are widely disputed, and several prominent theories currently hold sway (e.g., Blagosklonny, 2008). The oxidative stress theory of aging, first proposed by Harman (1956), is one of the most familiar yet beleaguered concepts in the study of physiological aging of animals. Under this “classical” oxidative stress theory of aging, damage inflicted by reactive oxygen species (ROS) to biomolecules within the cellular environment gradually accumulates, leading to the organisms' demise. This theory (actually more of a hypothesis) became rapidly focussed on mitochondria after these organelles were found to be an important source of ROS in the cell (Harman, 1972). In search of supporting evidence for this theory, and beginning in the 1970s, comparative and experimental studies have addressed a number of possible physiological correlates of longevity that are related to the amelioration of this oxidative stress. These include interspecies comparisons of constitutive levels of antioxidants (mostly cytosolic, reviewed in Hulbert, Pamplona, Buffenstein, & Buttemer, 2007) and membrane lipid composition for their susceptibility to oxidation (reviewed in Hulbert et al., 2007; Naudi et al., 2013; Valencak & Ruf, 2007). More recently, the “uncoupling to survive” hypothesis has been suggested as a means of reducing oxidative stress that can expand lifespan (Brand, 2000; Echtay & Brand, 2007; Speakman et al., 2004). Beginning in the 1990s, however, an influential series of studies compared the rate of hydrogen peroxide (H_2_O_2_) efflux from isolated mitochondria as a proxy of all ROS produced by this organelle. In early such studies, the underlying assumption was that the rate of mitochondrial ROS production would be the primary factor associated with longevity, after failure to associate cytosolic antioxidants to longevity (Barja, Cadenas, Rojas, Lopeztorres, & Perezcampo, 1994). Some later studies included measures of antioxidants again in a more encompassing view that considered the balance between production and elimination of ROS as being a key to longevity, but very few of them specifically interrogated a role for mitochondrial matrix antioxidants.

In the following decades, strong debate arose regarding the ideal choice of model species for comparative studies of longevity. Unequal body sizes and evolutionary distances between species have been pointed out as potential biases (i.e., allometric relationships and pseudoreplication, Garland & Adolph, 1994; Lambert et al., 2007; Speakman, 2005a, 2005b). Considerations of basal and maximal metabolic rates, and the validity of comparing between flying and nonflying species have also been debated as important aspects of the selection of model species (Rodriguez et al., 2011; Speakman et al., 2015). Parallel to the discussion of these important theoretical aspects, significant advances were made in our understanding of the mechanisms of mitochondrial ROS metabolism, primarily from rodent skeletal muscle, and to a lesser extent from other tissues. These evolving insights continue to change our understanding of mitochondrial ROS metabolism and have begun to raise questions regarding whether or not the means of measuring mitochondrial ROS production have in fact been a proximal limitation in this endeavor, and whether matrix (intramitochondrial) antioxidants play a unique but underappreciated role in this balance. Despite these emerging debates, several reviews have attempted to draw conclusions regarding the validity of the mitochondrial oxidative theory of aging, based to a large extent on findings from comparative studies of mitochondrial ROS formation rates (Barja, 2013; Buffenstein, Edrey, Yang, & Mele, 2008; Hekimi, Lapointe, & Wen, 2011a; Lambert & Brand, 2007; Robb, Christoff, Maddalena, & Stuart, 2014; Rodriguez et al., 2011; Stuart, Maddalena, Merilovich, & Robb, 2014). However, if the primary contributions measuring mitochondrial ROS formation rates are in fact flawed or incomplete in their consideration of mitochondrial ROS metabolism as a whole, and if matrix antioxidants have in fact been overlooked (or at least under‐considered), then a key piece of the puzzle may still be missing.

Approximating total mitochondrial ROS production by measuring the rate of H_2_O_2_ formation with the Amplex red system is primarily flawed as a direct or indirect consequence of the activity of matrix antioxidants capable of consuming H_2_O_2_ (Munro, Banh, Sotiri, Tamanna, & Treberg, 2016). The goals of this review are: (a) to critically consider what information may be derived from previous measurements of mitochondrial ROS formation rate using the horseradish peroxidase/probe detection systems, (b) to question if, in the case of comparative studies of longevity, this information is sufficient to draw conclusions regarding a putative relationship between evolutionary modulation of the rate of ROS formation by mitochondria in increasing longevity, and (c) to suggest alternative means of addressing the global questions of how the balance between ROS formation and elimination is modulated as a whole, and which component is modulated: formation of ROS or their elimination? Finally, we propose that we are not yet in a position to draw conclusions regarding the validity of the mitochondrial oxidative theory of aging, at least not based on the contributions of comparative studies of H_2_O_2_ formation rates from isolated mitochondria (see Table 1 for a list of studies).

**Table 1:** Frequency of respiratory substrates, inhibitors, and ADP usage in past comparative studies of longevity. Data are from the 18 studies that used a horseradish peroxidase‐based assay for monitoring mitochondrial H_2_O_2_ efflux. Except for glu + mal + succ, which elicits convergent electron flow, only substrate/inhibitor combinations used more than once were included. Also included is one study, not in the field of longevity, presenting similar data on species with diverging lifespan (Kuzmiak, Glancy, Sweazea, & Willis, 2012)

Respiratory substrates	No ADP	+ADP	Reference
*Pyruvate + malate*	6	1	Herrero and Barja (1997, 1998); Kuzmiak et al. (2012); Lambert et al. (2007); Montgomery, Hulbert, and Buttemer (2011); Montgomery et al. (2012)
*Pyruvate + malate + antimycin A*	3	0	Herrero and Barja (1997, 1998); Montgomery et al. (2012)
*Pyruvate + malate + antimycin A + myxothiazol*	2	0	Herrero and Barja (1997, 1998)
*Pyruvate + malate + rotenone*	3	0	Herrero and Barja (1997, 1998); Lambert et al. (2007)
*Glutamate + malate*	3	0	Brown, McClelland, Faure, Klaiman, and Staples (2009); Kuzmiak et al. (2012); Munro, Pichaud, Paquin, Kemeid, and Blier (2013)
*Succinate*	11	1	Barja, Cadenas, Rojas, Pérez‐Campo, and López‐Torres (1994); Barja and Herrero (1998); Csiszar et al. (2007); Ku, Brunk, and Sohal (1993); Lambert et al. (2007); Lambert et al. (2010); Montgomery, Hulbert, and Buttemer (,^2012^, ^2011^); Munro et al. (2013); Sohal et al. (1990); Sohal, Ku, and Agarwal (1993)
*Succinate + rotenone*	5	1	Barja and Herrero (1998); Brown et al. (2009); Lambert et al. (2007); Lambert et al. (2010); Montgomery et al. (2011); Munro et al. (2013)
*Succinate + antimycin A*	3	0	Barja and Herrero (1998); Ku and Sohal (1993); Montgomery et al. (2012)
*Succinate + rotenone + antimycin A*	2	0	Brown et al. (2009); Robert, Brunet‐Rossinni, and Bronikowski (2007)
*Glutamate + malate + succinate*	1	1	Munro et al. (2013)

## 2 WHAT DOES TRADITIONAL H_2_O_2_ EFFLUX ASSAYS REALLY MEASURE?

**Figure 1:**
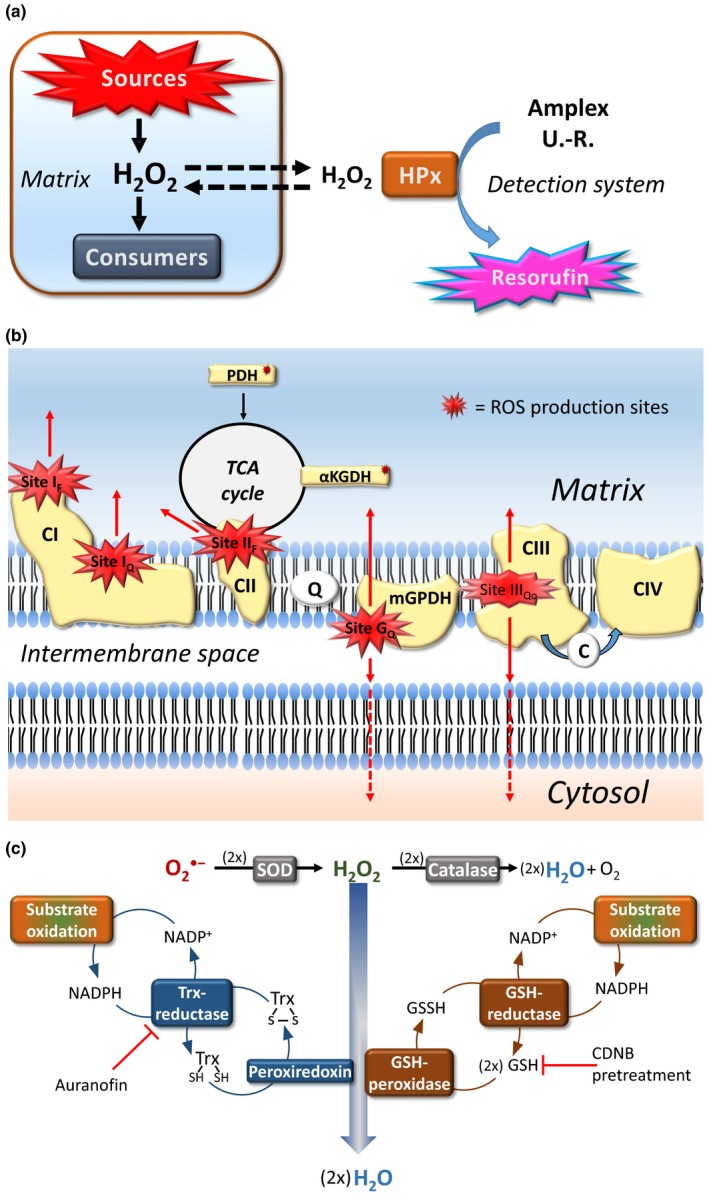
(a) Traditional horseradish peroxidase‐based H_2_O_2_ detection system. Amplex UltraRed is shown as a probe, but multiple other probes have also been used, including homovanillic acid. (b) Superoxide/H_2_O_2_ formation sites of primary importance. Red arrows indicate the topology of superoxide/H_2_O_2_ release. (c) Antioxidant enzymatic pathways of the mitochondrion. Abbreviations: Amplex U.‐R., Amplex UltraRed; C, Cytochrome C; CDNB, 1‐chloro‐2,4‐dinitrobenzene; CI, complex I; CII, complex II; CIII, complex III; CIV, complex IV; GSH, glutathione; HPx, horseradish peroxidase; mGPDH, mitochondrial sn‐glycerol 3‐phosphate dehydrogenase; PDH, pyruvate dehydrogenase; Q, ubiquinone; SOD, superoxide dismutase; Trx, thioredoxin; αKGDH, alpha‐ketoglutarate dehydrogenase. Panels a and c adapted with permission from Munro and Treberg (2017)

Measuring the rate at which mitochondria release H_2_O_2_ in an assay medium would be an acceptable means of estimating total ROS formation by the mitochondrion if it was not for the partial internal consumption of mitochondrially derived ROS (Figure 1a). Most mitochondrial ROS are initially formed as superoxide anion (O_2_^•^ **^⁻^**, hereafter referred to as superoxide), with some contribution of direct H_2_O_2_ production, and negligible production of hydroxyl radical (OH^•^; Brand, 2016; Wong, Dighe, Mezera, Monternier, & Brand, 2017). Superoxide produced inside (the majority) and outside the mitochondrion spontaneously dismutes into the more chemically stable H_2_O_2_ or is rapidly converted to H_2_O_2_, respectively, by the mitochondrial endogenous superoxide dismutase (MnSOD or SOD2) and the cytosolic form (Cu/ZnSOD or SOD1). Past studies working with isolated mitochondria have used a detection system that is composed of a fluorescent probe (e.g., homovanillic acid, Amplex Red^®^, and more recently Amplex UltraRed^®^), and the enzyme horseradish peroxidase, which catalyses oxidation of the probe, thereby extinguishing or sparking its fluorescence, depending on the nature of the probe. The absence of an electronic charge on H_2_O_2_, and the presence of aquaporins (Bienert & Chaumont, 2014), allows H_2_O_2_ molecules to diffuse across the inner membrane to reach the detection system in the reaction medium (efflux). This flux is important because the horseradish peroxidase cannot cross biological membranes; hence, the detection of H_2_O_2_ only occurs outside the mitochondrion (Figure 1a), even though the Amplex UltraRed probe can cross the inner membrane (Miwa et al., 2016).

Past studies relying on this method for the measurement of mitochondrial ROS formation assumed that internal consumption of H_2_O_2_ by matrix‐based antioxidants was negligible (Barja & Herrero, 1998; Herrero & Barja, 1998), probably because the affinity of the probes for H_2_O_2_ is high (mostly after introduction of homovanillic acid and more so after introduction of Amplex Red and Amplex UltraRed). Mitochondria, however, possess three types of matrix antioxidants capable of consuming H_2_O_2_. In mitochondria of most tissues investigated so far, the two most important are the glutathione (GSH)‐ and thioredoxin (Trx)‐dependent enzymatic pathways that involve, respectively, the GSH‐peroxidase (GPx 1&4) and the peroxiredoxins (Prx 3&5; Lu & Holmgren, 2014; Figure 1c). The activity of these antioxidant pathways requires the provisioning of reducing equivalents in the form of NADPH. Providing respiratory substrates to the mitochondrion leads to the production of NADH and NADPH, and the exchange of NADH for NADPH. Importantly, provision of substrate thus elicits both the consumption and the production of ROS simultaneously (Treberg, Munro, Banh, Zacharias, & Sotiri, 2015). Equally importantly, it has been shown in rat skeletal muscle and heart mitochondria that maximal rates of H_2_O_2_ consumption by matrix antioxidants are obtained with much lower concentrations of respiratory substrates than those required for obtaining maximal rates of superoxide/H_2_O_2_ production, irrespective of the type of respiratory substrate (Munro & Treberg, 2017; Treberg et al., 2015). Hence, we now know that any substantial formation of H_2_O_2_ will inevitably be accompanied by total activation of mitochondrial pathways for its consumption.

A simple means of determining whether mitochondria from a given tissue possess a greater maximal capacity for the production of H_2_O_2_ than for its consumption is to provide respiratory substrates at the same time as adding a saturating [H_2_O_2_] in the medium (Drechsel & Patel, 2010; Munro et al., 2016; Starkov et al., 2014). If the net outcome of the assay is a decline in [H_2_O_2_] in the respiration medium, then the maximal rate of consumption is higher than the rate of formation for a given combination of respiratory substrates tested. Zoccarato, Cavallini, and Alexandre (2004) provided early evidence that, for most substrates, the maximal capacity of (energized) mitochondria to consume H_2_O_2_ was several times higher than their maximal capacity for its formation. More precisely, recent studies showed that, for mouse brain, heart, and skeletal muscle mitochondria, H_2_O_2_ consumption exceeds production by 1–12 nmol min^−1^ mg protein^−1^, depending on the substrates (Banh & Treberg, 2013; Munro et al., 2016). In comparison, rates of H_2_O_2_ efflux obtained with traditional assays typically range between 0.1 and 3 nmol min^−1^ mg protein^−1^ for the same mitochondria/substrates (Goncalves, Quinlan, Perevoshchikova, Hey‐Mogensen, & Brand, 2015; Munro et al., 2016). Notably, the liver is a unique case because, in this tissue, catalase is the main consumer of H_2_O_2_. Consumption of H_2_O_2_ by rodent liver mitochondria is particularly rapid in saturating levels of H_2_O_2_, ranging from 40 to 120 nmol min^−1^ mg protein^−1^ (Drechsel & Patel, 2010; Lopert & Patel, 2014, unpublished results D. Munro and J. Treberg). This reflects the high catalytic capacity of this enzyme in the presence of high [H_2_O_2_]. Critically, the finding that all mitochondria investigated so far are potentially capable of consuming H_2_O_2_ faster than they produce it, under many different substrate conditions, recently raised a warning flag regarding the use of traditional H_2_O_2_ efflux assays for estimating the true rate of ROS formation from isolated and intact mitochondria. Internal consumption of H_2_O_2_, before its diffusion to the reaction medium, is actually much higher than originally anticipated.

**Figure 2:**
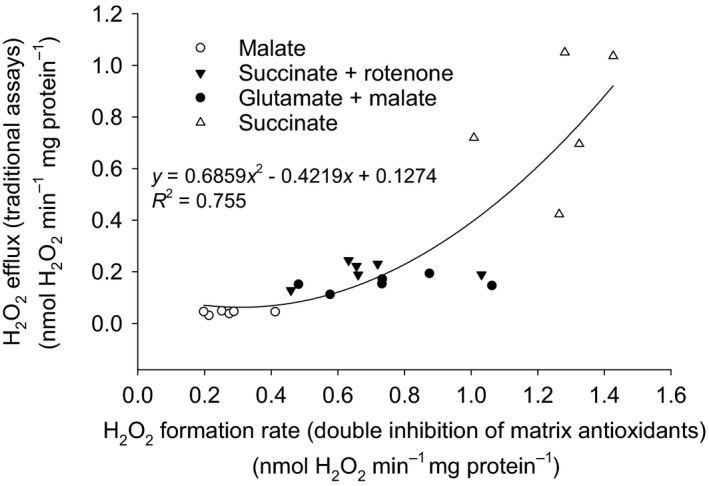
Rat skeletal muscle mitochondrial H_2_O_2_ efflux as a function of actual H_2_O_2_ formation rate. Efflux is defined as the proportion of H_2_O_2_ that can escape consumption by matrix antioxidants and reach the detection system after diffusion across the inner membrane. Actual H_2_O_2_ formation rate (*x*‐axis) is measured after inhibition of matrix antioxidants

To probe this concern, our group continued in the line of Treberg and coworkers (Treberg, Quinlan, & Brand, 2010), and validated inhibition approaches for compromising both the GSH‐ and Trx‐dependent pathways in rodent skeletal muscle (see Figure 1c). First, auranofin, when added to the reaction medium, will inhibit the thioredoxin reductase, thereby compromising the activity of the peroxiredoxins. Secondly, pretreatment of isolated mitochondria with 1‐chloro‐2,4‐dinitrobenzene (CDNB) sequesters GSH in the matrix, therefore compromising the activity of the glutathione peroxidases without side effects on the rate of ROS formation, as with direct addition (Liu, Fiskum, & Schubert, 2002). With rat skeletal muscle, this combination of inhibitory approaches allowed for (almost) complete elimination of the internal consumption of H_2_O_2_, because catalase activity is negligible in this tissue. Comparing results derived from the use of traditional H_2_O_2_ efflux assays (using rat skeletal muscle mitochondria) with or without inhibition of their matrix antioxidant pathways, we found that as much as 43%–84% of the total H_2_O_2_ formed inside mitochondria could be consumed before detection using traditional assays (Figure 2, Munro et al., 2016). These results definitively contradicted the inherent (or explicit in some cases) assumption in the field that only minor to negligible underestimation of actual H_2_O_2_ formation rates occurs with the use of traditional (horseradish peroxidase‐based) efflux assays. Given that these assays had been used for three decades in comparative studies investigating a possible negative relationship between longevity and mitochondrial ROS formation rate, this finding raises the concern that any relationship found was in fact a positive relationship between longevity and the rate of matrix H_2_O_2_ scavenging capacity; a critical distinction that these studies could unfortunately not make.

## 3 IS THE TRADITIONAL H_2_O_2_ EFFLUX ASSAY CAPABLE OF ADDRESSING THE MITOCHONDRIAL OXIDATIVE THEORY OF AGING?

Hydrogen peroxide efflux measured using traditional assays may be more accurately considered as the net H_2_O_2_ production minus the proportion consumed in the matrix. Efflux of H_2_O_2_ can thus be argued to represent a proxy of the in vivo mitochondrial contribution to cytosolic H_2_O_2_. As such, the traditional approach for measuring H_2_O_2_ efflux is acceptable for addressing the “classical” mitochondrial oxidative theory of aging, according to which aging results from the accumulation of oxidative damage to cellular macromolecules in general.

Unfortunately, the contextual framework of the theory has changed since its inception. There is now ample evidence that the oxidation of renewable cytosolic macromolecules such as proteins is not directly related to longevity (Andziak, O'Connor, & Buffenstein, 2005; Hekimi et al., 2011a; Lewis, Andziak, Yang, & Buffenstein, 2013; Stuart et al., 2014). Instead, the debate has now shifted to the question of whether or not oxidation of mitochondrial DNA (mtDNA), or other permanent damage to mitochondria, represents an important contribution to the process of senescence (Barja, 2013; Dai, Chiao, Marcinek, Szeto, & Rabinovitch, 2014; Kukat & Trifunovic, 2009; Shabalina et al., 2017), and if this effect could thus be modulated to extend longevity. Within this revised context, it could nonetheless be argued that the balance between the rates of H_2_O_2_ formation and consumption inside the matrix is represented by H_2_O_2_ efflux. This is because efflux (diffusion outside the mitochondrion) is directly proportional to matrix [H_2_O_2_]. In other words, even though values derived from efflux assays are not absolute rates of H_2_O_2_ formation, they would allow the ranking of species with respect to their susceptibility to mtDNA oxidation based upon differences in resulting matrix [H_2_O_2_]. The major and most obvious flaw in this rationale is that, by its very nature, this measure is blind to the underlying mechanisms that may explain differences across species. Do interspecies differences in lifespan correlate with rates of H_2_O_2_ production or with rates of internal (matrix) H_2_O_2_ consumption? At the minimum, this is not a negligible piece of information in the quest to develop a complete understanding of how oxidative processes contribute to aging. Notwithstanding this major limitation, the caveats of only measuring H_2_O_2_ efflux are unfortunately much more pervasive when it comes to the context of comparative studies of animal longevity, as will be discussed in the following sections.

## 4 THE PROPORTION OF H_2_O_2_ EXPOSED TO MATRIX ANTIOXIDANTS CAN VARY BY SPECIES

There are at least 10 sites of superoxide/H_2_O_2_ production in mitochondria, with the quantitatively more important being located inside the inner membrane (mostly sites I_F_, I_Q_, II_F_, III_Qo_) and the matrix, including enzymes of the TCA cycle (Figure 1b; see Goncalves et al., 2015; Wong et al., 2017), for a more comprehensive description). As we shall see, there is likely considerable variation between species in the activity and function of most of these sites, and thus, integrating all of these variables within interspecies comparisons is not trivial. For rat skeletal muscle, site III_Qo_ has the highest maximal capacity in the presence of saturating substrate (succinate) concentrations and inhibitors. Site III_Qo_ is particularly important in this consideration as it not only releases superoxide inside the mitochondrion (matrix side), but also to the intermembrane space. Early estimations suggested that the large majority of the superoxide produced by site III_Qo_ is released to the intermembrane space (St‐Pierre, Buckingham, Roebuck, & Brand, 2002). During traditional efflux measurements, superoxide released to the intermembrane space will immediately react with the exogenously added SOD, added as one of the assay reagents. The H_2_O_2_ produced by this reaction will then immediately react with the detection system, which possesses a much higher affinity for H_2_O_2_ than do the antioxidants present in this compartment. Thus, as opposed to the case with matrix‐borne H_2_O_2_, there is no partial consumption of the H_2_O_2_ released to the intermembrane space. A bias with comparing species, when site III_Qo_ superoxide production is activated, will therefore arise if the relative proportion of H_2_O_2_ originating from this site varies between species.

No direct interspecific comparisons of the relative contribution of site III_Qo_ to total superoxide/H_2_O_2_ formation have been made yet, but indirect evidence suggests it could be species‐specific. The relative contribution of site III_Qo_ is very important after adding rotenone to succinate‐energized mitochondria and accounts for almost all superoxide/H_2_O_2_ formation if antimycin A is also added. There are some experimental examples wherein the addition of rotenone to succinate‐energized mitochondria has remarkably different effects on H_2_O_2_ efflux, suggesting a different relative or absolute contribution from site III_Qo_ between species. For instance, when comparing the pigeon and the rat, the addition of rotenone to succinate‐energized heart mitochondria reduced efflux by ~50% for the rat, whereas an increase was observed for the pigeon (Herrero & Barja, 1997). A similar discrepancy in the effect of rotenone addition was seen for brain mitochondria (Barja & Herrero, 1998). In addition, inconsistent effects of the addition of rotenone and antimycin A have been reported for succinate‐energized liver mitochondria of various species of endotherms (Sohal, Svensson, & Brunk, 1990). These examples suggest we should expect large variations in the relative proportion of H_2_O_2_ to total H_2_O_2_ formation, accounted for by site III_Qo_ across species.

Previous investigations of the topology of superoxide release at site III_Qo_ suggest the proportion directed to the intermembrane space is ~35% in mouse skeletal muscle (Muller, Liu, & Van Remmen, 2004), which is slightly different from another rodent (rat), wherein it was estimated to represent 47% (Treberg et al., 2010). In contrast, only 25%–30% of superoxide was found to be released to the intermembrane space in another evolutionary lineage: in *drosophila* mitochondria (Miwa & Brand, 2005; Miwa, St‐Pierre, Partridge, & Brand, 2003). These examples suggest we should expect large variation in the relative proportion of H_2_O_2_ formed by site III_Qo_ that is released to either the matrix side or the intermembrane side across species.

Whether a different proportion of total H_2_O_2_ formation originates from site III_Qo_ between species, or a different proportion of the production from this site is released to the matrix side, in both cases, a different proportion will be exposed to matrix antioxidants, creating a bias in the proportion of H_2_O_2_ being consumed by matrix antioxidants. This bias is maximized when succinate and rotenone are used (with or without antimycin A), an experimental condition that has been often used in past studies (Table 1).

More recently, another ROS producing site (G_Q_, glycerol phosphate dehydrogenase) has also been found to release ~50% of its superoxide to the intermembrane space in rat skeletal muscle mitochondria (Orr, Quinlan, Perevoshchikova, & Brand, 2012). Although the contribution of G_Q_ was previously thought to be negligible, this site is now estimated to account for about 1/5th of the total H_2_O_2_ formed during exercise in rat skeletal muscle mitochondria (Goncalves et al., 2015). For this superoxide/H_2_O_2_ formation site, marked differences in the maximal generation capacity have been observed at least across tissues for the rat (Brand, 2016; Orr et al., 2012). Studies comparing the relative contribution of this ROS formation site across species are warranted.

Taken together, it is clear that if measurements of efflux (no inhibition of matrix antioxidants) are used to estimate the balance between formation and elimination of H_2_O_2_ by mitochondria, these estimates must accept a potentially large interspecific bias when succinate is used as a substrate, and possibly also with glycerol‐3‐phoshate.

## 5 SUBSTRATE COMBINATIONS WERE SELDOM REPRESENTATIVE OF THE IN VIVO MILIEU

Past studies have predominately utilized combinations of substrates/inhibitors that are less relevant to in vivo metabolism. The primary reasons for these choices may have been that (a) the high rates of superoxide/H_2_O_2_ formation with succinate were assumed to incorporate the bulk of the mitochondrial contribution to overall ROS metabolism, and (b) rates of superoxide/H_2_O_2_ formation for relevant conditions might have been very difficult to detect when matrix antioxidants had been allowed to consume H_2_O_2_ during the assay.

**Figure 3:**
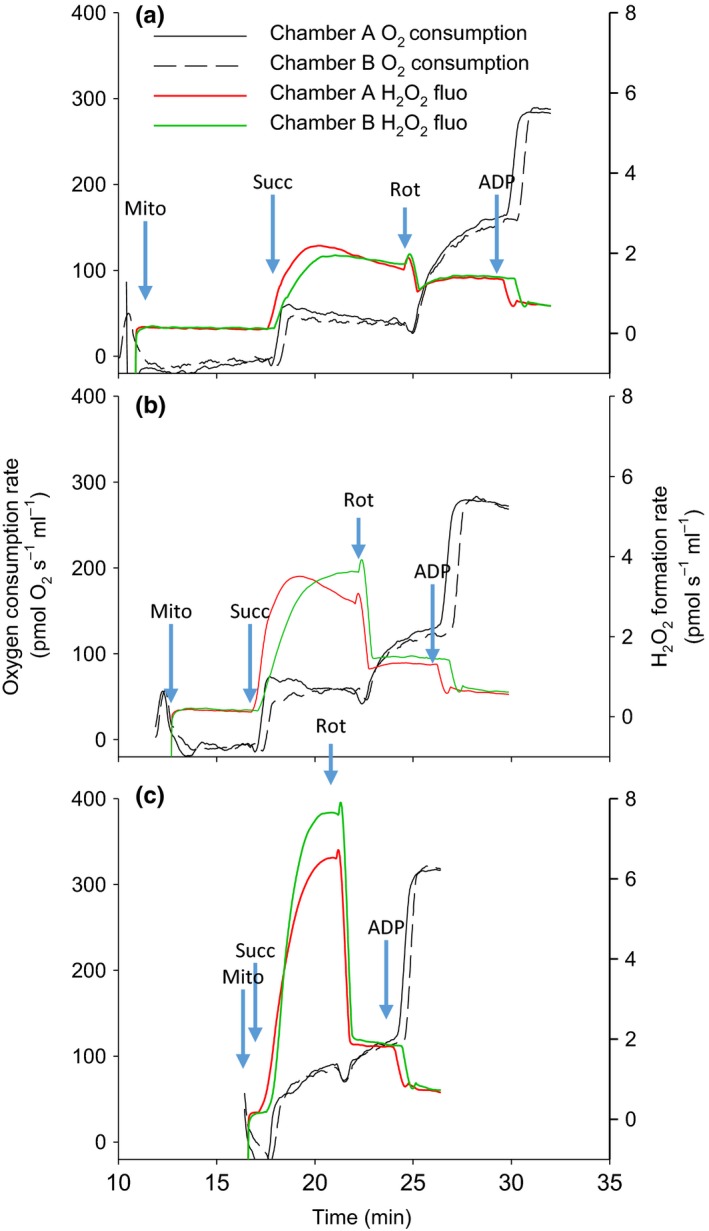
Representative traces of oxygen consumption and H_2_O_2_ formation rates (fluorescence) in the Oroboros O2K by murine skeletal muscle mitochondria. Panels a–c represent consecutive measures with the same mitochondrial preparation and a constant 0.08 mg/ml mitochondrial protein. Succinate has been added 7, 4, and 0.5 min after sealing the recording chambers, respectively, for a, b, and c. Note the profound decrease in superoxide/H_2_O_2_ formation rate during succinate oxidation (production dominated by site I_Q_), with increasing incubation time before addition of succinate. This effect is not seen for the rate obtained after the addition of rotenone (production dominated by site III_Qo_). No such decrease in succinate‐supported superoxide/H_2_O_2_ formation rate with incubation time was observed for NMR skeletal muscle mitochondria (not shown)

The use of succinate as a substrate generally supports the highest rates of ROS formation in vertebrates, especially when antimycin A is added. However, the work by Goncalves et al. (2015) showed how much absolute rates and sites of superoxide/H_2_O_2_ formation differ from those obtained with this substrate as we tend toward conditions better mimicking in vivo. Using isolated rat skeletal muscle mitochondria, the authors measured superoxide/H_2_O_2_ formation in conditions mimicking “rest,” “mild,” and “intense” physical activity. This was achieved by using almost all substrates and effectors (including ADP) of mitochondrial respiration simultaneously at concentrations found in vivo for the corresponding degree of physical activity. An important conclusion from this work is that for four sites of superoxide formation, the individual maximal rate of formation (with saturating substrates and inhibitors) was higher than the combined production of all ten sites during any of the three conditions mimicking the in vivo milieu (Goncalves et al., 2015, see Figure 3).

Perhaps the greatest pitfall with succinate is that it does not elicit superoxide formation at physiologically relevant sites. Succinate is particularly effective at elevating superoxide formation at sites I_Q_, II_F_, and III_Qo_, respectively, found in complexes I, II, and III (Brand, 2010; Goncalves et al., 2015). Production at site I_Q_ during succinate oxidation is directly involved in ischemia/reperfusion injury (Chouchani et al., 2016) and may have unique relevance to cell signaling (Scialo, Fernandez‐Ayala, & Sanz, 2017). However, in a breakdown of the relative contribution of all ten sites to overall superoxide/H_2_O_2_ formation for the three in vitro conditions mimicking in vivo, the study from Goncalves et al. (2015) shows that sites activated by succinate are only important contributors during “rest.” Instead, site I_F_ previously thought of as having negligible importance to overall mitochondrial ROS production, predominates during mild to intense physical exercise. Formation of superoxide/H_2_O_2_ by site I_F_ is directly linked to the redox state of the mitochondrial NAD(P)H pool (Brand, 2010; Goncalves et al., 2015), which is more reduced with substrates such as pyruvate, glutamate, and malate. Comparing species for conditions eliciting ROS formation at site I_F_ therefore requires the use of substrates for complex I.

The higher relative contribution of site I_F_ during mild and intense activity further pertains to the increase in ADP concentrations used to reflect physical activity in muscles. Sites I_Q_ and III_Qo_ of complexes I and III may have very high maximal superoxide/H_2_O_2_ formation rates, but they are only important contributors so long as the protonmotive force is high (i.e., LEAK state). A minor decrease in the protonmotive force, as occurs after addition of subsaturating [ADP], will immediately decrease superoxide/H_2_O_2_ formation at these sites (Goncalves et al., 2015; Lambert & Brand, 2004). In contrast, adding physiological [ADP] will only have limited effect on the reduction state of the NAD(P)H pool, and thus limited effect in decreasing the contribution from site I_F_. Comparing species for their capacity to produce superoxide at site I_F_ is thus best achieved by using ADP.

Which site of superoxide/H_2_O_2_ formation should be compared between species is a difficult question to answer and may depend on the choice of tissue. It can be argued that for the heart, brain, and liver, which are under constant demand for ATP, site I_F_ may be paramount. In contrast, the skeletal muscle is at rest for significant portions of the overall lifespan in many species, during which case sites I_Q_ and III_Qo_ would be relevant also. These sites may be particularly important for skeletal muscle aging in species experiencing large fluctuations in physical activity. Markers of oxidative stress are known to increase during or immediately after sustained intense physical activity, such as migratory flight (Jenni‐Eiermann, Jenni, Smith, & Costantini, 2014). From a mechanistic point of view, it is well known that increased demand for ATP (increased ADP) does not translates into higher rates of superoxide/H_2_O_2_ formation with isolated mitochondria (Lambert & Brand, 2004; Munro & Treberg, 2017). An oxidative stress may rather occur during the short transition period immediately after cessation of physical activity—for example, it may occur in the minutes after catching free‐ranging individuals, just before biopsy sampling. If recruitment of the dehydrogenases of the TCA cycle occurs in response to sustained physical activity (e.g., through post‐translational modifications), a temporary surge in the provisioning of electrons to the ETS enzymes may occur briefly during abrupt transition to a “rest state,” when [ADP] rapidly decreases. During this short period, the conditions would be created for important superoxide/H_2_O_2_ formation at sites I_Q_ and III_Qo_. Repeated cycles of these events, when the antioxidant defenses are temporarily overwhelmed, may lead to skeletal muscle aging. Therefore, it may be worth measuring superoxide/H_2_O_2_ formation rate in multiple energetic conditions when comparing species in general, and especially for the skeletal muscle.

Unfortunately, many past studies heavily relied on succinate (with or without inhibitors) for comparing mitochondria of the heart, brain, and liver, which are under sustained demand for ATP in vivo. Using substrates for complex I (and ADP), which would have elicited superoxide/H_2_O_2_ formation at site I_F_, might have been avoided experimentally simply because of the low rates of superoxide/H_2_O_2_ generated. Our work with rat skeletal muscle mitochondria might explain why rates of H_2_O_2_ efflux are so low in these conditions. We have found that the less superoxide/H_2_O_2_ mitochondria actually produce, the greater is the percentage of H_2_O_2_ consumed by matrix antioxidants (Figure 2, Munro et al., 2016). For example, in the absence of ADP, succinate produces superoxide/H_2_O_2_ at high rates and the underestimation of total ROS formation by the Amplex system was only 43%. At the other extreme, malate produces very little superoxide/H_2_O_2_, and the underestimation reached 84%. Such a loss of signal, when the absolute rate of superoxide/H_2_O_2_ formation is already very low, may have been especially problematic with early generations of probes that had a lower affinity for H_2_O_2_.

Using homovanillic acid as a fluorescent probe (which is already an improvement over earlier probes), St‐Pierre et al. (2002) measured H_2_O_2_ efflux from heart and skeletal muscle mitochondria fueled with pyruvate and malate, and referred to the rates of efflux as being “very low.” As estimated from their figure, rates for these substrates were about 0.01 nmol H_2_O_2_ min^‐1^ mg protein^‐1^, which in our experience, is quite low relative to background. Using the same probe for monitoring H_2_O_2_ efflux from lovebird heart and skeletal muscle mitochondria supplemented with pyruvate + malate, Montgomery, Hulbert, and Buttemer (2012) reported undetectable rates, unless the inhibitor antimycin A was added. Antimycin A is known to greatly increase superoxide formation at site III_Qo_ of complex III. In these two studies, pyruvate was used in combination with malate, which markedly elevates the rates of superoxide/H_2_O_2_ formation as compared to malate alone. In contrast, we measured rates of H_2_O_2_ formation with malate alone after inhibiting matrix consumption of H_2_O_2_ in rat skeletal muscle mitochondria, and we observed a rate of 0.3 nmol H_2_O_2_ min^−1^ mg protein^−1^, which is well above instrumental background (Munro et al., 2016). Inhibiting matrix antioxidants thus supports comparing between species with confidence, even for very low rates of superoxide/H_2_O_2_ formation. This provides new capabilities to investigate mitochondrial ROS in more physiological conditions.

Lastly, it is unfortunate that past studies almost never presented data obtained in “convergent electron flow,” whereby electrons are fed to the Q‐pool by complex I at the same time as by complex II (or glycerol phosphate dehydrogenase). The implications of only having electrons flowing through “chains” of complexes, instead of a “system,” are difficult to assess. Complex III can be found in isolation, but is most often found associated as supercomplexes I_n_ + III_n_ + IV_n_ and III_n_ + IV_n_ (Lapuente‐Brun et al., 2013). Importantly, the type of fuel substrate is suggested to change the proportion of complex III found under each type of association versus the free form (Lapuente‐Brun et al., 2013). To our knowledge, nothing is known of the influence of each type of association, nor regarding possible electron channeling on the rate and topology of superoxide/H_2_O_2_ formation by complex III, and especially by site III_Qo_.

Table 1 summarizes the energetic conditions used in past studies. It is clear that, very often, the conditions selected were those favoring high rates of superoxide/H_2_O_2_ formation at the level of site I_Q_ and III_Qo_ (i.e., succinate with or without inhibitors, and in the absence of ADP).

## 6 OTHER BIAS IN MEASURING ROS PRODUCTION IN THE CONTEXT OF COMPARATIVE STUDIES OF LONGEVITY

The rate of superoxide/H_2_O_2_ formation has been shown to decline with time after isolation of mitochondria from rat skeletal muscle. This effect seems to primarily involve superoxide production at site I_Q_ as it is observed in the presence of succinate as the sole respiratory substrate, but absent when rotenone is added (Treberg, Braun, Zacharias, & Kroeker, 2018). Preliminary tests for our recent study (Munro, Baldy, Pamenter, & Treberg, 2019) showed that the time spent (in the absence of substrates) by mice skeletal muscle mitochondria inside the chambers of the Oroboros, before addition of substrates, also leads to a pronounced decline in the capacity for superoxide/H_2_O_2_ generation at site I_Q_ (succinate alone, Figure 3). This effect is however not seen in the presence of glutamate + malate and is also not observed in mitochondria of the long‐lived naked mole‐rat (NMR, not shown in Figure 3). Note that mice have been used in at least seven studies, beginning in 1990, as a reference species with which to compare various mammalian longevity models; the methodology of these studies rarely mentions if substrates were added immediately or after a stabilization period when mitochondria are in the instrument. A rapid deterioration of the superoxide/H_2_O_2_ generation capacity of site I_Q_ in various conditions may characterize short‐lived species as compared to long‐lived ones, the latter having been shown to possess a greater general robustness of their proteome (Pérez et al., 2009; Treaster et al., 2014). This raises the concern that past studies might have underestimated superoxide/H_2_O_2_ formation rates of short‐lived species. In our case, rates of superoxide/H_2_O_2_ formation were always lower for mice when a five minutes of equilibration period was observed before addition of succinate (*n* = 3), whereas acquiring data immediately after addition of mitochondria to the chambers resulted in significantly higher rates for mice as compared to NMR (Munro et al., 2019).

## 7 ADDITIONAL FUNDAMENTAL ISSUES WITH MITOCHONDRIAL H_2_O_2_ EFFLUX

In addition to potential bias specifically associated with longevity studies, there are a number of fundamental issues with the measure of H_2_O_2_ efflux that has seldom been accounted for in previous comparative studies of longevity or aging. These caveats are only briefly explained here since they have been more thoroughly discussed in the references provided below.

First, the carboxylesterases present in liver mitochondria, and to a lesser extent in kidney mitochondria, can convert Amplex UltraRed to resorufin (i.e., the fluorescent form of the probe). This conversion can occur at high rates in the absence of H_2_O_2_, unless the inhibitor phenylmethylsulfonyl fluoride (PMSF) is used (Miwa et al., 2016). The rate of this reaction increases with chronological age (and decreases under caloric restriction), which may have led to the conclusion that mitochondrial production of H_2_O_2_ increases with age when using this tissue (Miwa et al., 2016, supp. Material figure 8).

Second, α‐ketoacids such as pyruvate auto‐oxidize H_2_O_2_ into water. When pyruvate is added in concentrations close to those typically used for energizing mitochondria (2 mM), important direct scavenging of H_2_O_2_ is observed (Desagher, Glowinski, & Prémont, 1997).

Third, the fluorescent probe Amplex UltraRed was shown to have an inhibitory effect on mitochondrial respiration with permeabilized cells, even at concentrations (30 µM) below the manufacturer's recommendations (50 µM; Makrecka‐Kuka, Krumschnabel, & Gnaiger, 2015). In support of this conclusion, we observed an average 13% loss of respiration with malate and a 10% loss with glutamate + malate + succinate + ADP, when using 50 µM Amplex UltraRed during pilot tests with mice skeletal muscle mitochondria (data not shown). Preliminary tests with 10 µM Amplex UltraRed revealed that this concentration was sufficient to maintain the linearity of standard curves, and allowed us to avoid adverse effects on the energetics of isolated mitochondria (Munro et al., 2019).

Finally, the affinity of cytochrome C oxidase for oxygen seems much higher than that of important ROS production sites, particularly for site I_Q_ (Treberg et al., 2018). This means that the rate of H_2_O_2_ formation will diminish before (below 80 µM molecular oxygen) oxygen consumption rates significantly decline (Treberg et al., 2018). This whim of mitochondrial metabolism can be an issue when the fluorescence assay is conducted in closed chambers so as to monitor oxygen consumption in parallel, for example, as is commonly done with an oxygraph Oroboros 2K.

## 8 INTEGRATED H_2_O_2_ SCAVENGING CAPACITIES CAN ONLY BE MEASURED IN FUNCTIONAL MITOCHONDRIA

A number of previous comparative studies of longevity included measures of the activity of mitochondrial‐specific antioxidant enzymes in an attempt to describe “the other side of the equation”—that is, the capacity of mitochondria to detoxify their internally produced ROS. Measures of MnSOD are valid in this context, but the bulk of oxidative damage is believed to result from later steps of the Haber–Weiss reactions (after the formation of H_2_O_2_), leading to the formation of highly reactive hydroxyl radicals. Therefore, eliminating H_2_O_2_ is important in order to alleviate damages resulting from this suite of enzymatically catalyzed or spontaneous reactions. Measurements of enzymes involved in detoxifying H_2_O_2_ have unfortunately been incomplete in previous comparative studies of longevity, and thus do not adequately support comparisons between species based on their integrated capacity. Firstly, in most studies, only the activity of one of the peroxidases of the two redundant pathways has been measured (e.g., the glutathione peroxidase). This provides insufficient information because the relative contribution of each pathway varies profoundly across tissues and between species. For instance, the Trx‐dependent pathway accounts for over 80% of H_2_O_2_ consumption in isolated mice brain mitochondria (Drechsel & Patel, 2010), whereas the GSH‐dependent pathway dominates the H_2_O_2_ scavenging capacity of isolated skeletal muscle mitochondria in NMRs (see the section “the NMR vs. the mouse: a test case of a novel approach,” below). A few studies attempted to correct for the internal consumption of H_2_O_2_ using a model based on Treberg et al. (2010); however, this model only takes into account the GSH‐dependent pathway and is only valid for rat skeletal muscle mitochondria, within which this approach was developed.

Secondly, these two pathways function as a chain of enzymes originally fueled by the oxidation of respiratory substrates by the mitochondrion (Figure 1c). Many enzymatic steps are involved in setting the pace of activity of the entire pathway. Currently, very little is known regarding the relative flux control exerted at each step of these pathways in vivo. For example, the reductases could set a greater limitation in the flux of reducing equivalents than the peroxidases. Moreover, the conversion of NADH into NADPH by the nicotinamide nucleotide transhydrogenase (NNT) appears central to setting the level of activity of the reductases in some tissues and species (Dey, Sidor, & O'Rourke, 2016), and the activity of this enzyme is dependent on the protonmotive force (Lopert & Patel, 2014; Mailloux, 2018). The integrity of this complex suite of enzymatic and bioenergetics steps is lost along with the integrity of the inner membrane after freezing of the mitochondrial isolate. Instead, a very straightforward and much better proxy of the integrated capacity for the detoxification of H_2_O_2_ in vivo is to challenge energized isolated mitochondria with a bolus of H_2_O_2_ in a respiration medium, so as to follow the rate of H_2_O_2_ decay over time (Munro et al., 2019, 2016; Starkov et al., 2014; Treberg et al., 2010; Zoccarato et al., 2004). In this regard, we have shown for rat skeletal muscle mitochondria that malate is sufficient to spark full activity of both the GSH‐ and TRx‐dependent pathways while minimizing concomitant superoxide/H_2_O_2_ formation, providing an interesting proxy of the maximal capacity of the mitochondrion to consume H_2_O_2_ (Treberg et al., 2015). Among past studies, only that of Lambert, Buckingham, Boysen, and Brand, (2010) tested the H_2_O_2_ removal capacity of isolated mitochondria to determine whether any difference in the rate of efflux could be explained by matrix antioxidants. Unfortunately, no substrates were provided to the (pigeon and rat) heart mitochondria, precluding the GSH‐ and Trx‐dependent pathways from contributing to the elimination of H_2_O_2_.

## 9 CAN WE DRAW CONCLUSIONS FROM PAST STUDIES?

The pioneers of comparative studies of longevity addressed a fundamental question of biology that needed to be answered, but were not aware of many pervasive pitfalls and caveats associated with the measurement of mitochondrial ROS formation. To summarize, if we doubt conclusions drawn when (a) using succinate (incorrect sites of ROS formation for heart, brain, and liver, bias at site III_Qo_ related to the topology of superoxide release, bias at site I_Q_ related to the sensitivity to time elapsed after isolation or in the instrument, and sensitivity to oxygen partial pressure), (b) when using liver mitochondria with Amplex UltraRed (oxidation of Amplex Red by the carboxylesterases), (c) when drawing conclusions regarding heart and brain mitochondrial ROS efflux without testing the addition of ADP, and (d) when using pyruvate, which directly consumes H_2_O_2_, the little data remaining are still afflicted by an underestimation in the main measurement output (i.e., ROS efflux) ranging between 50% and 80%. If, in addition, we eliminate (e) all work not re‐investigated for bias of unequal body masses and unequal evolutionary distances (Lambert et al., 2007; Speakman, 2005b), the database on the relationship between longevity and mitochondrial ROS production is much less convincing. Nevertheless, it remains interesting that a negative relationship between efflux and longevity was sometimes found after re‐analyzing data for body sizes and evolutionary distances (Lambert et al., 2007), or when the chosen species naturally minimize these problems. Such negative relationships should be an indicator (albeit a partly biased one) that either the mitochondrial superoxide/H_2_O_2_ formation rate is lower in long‐lived species or that they possess greater mitochondrial H_2_O_2_ detoxifying capacities. Therefore, re‐assessing strong biogerontology models would be very interesting. For this purpose, however, novel methodological approaches are needed that are described below.

## 10 REDIRECTING FUTURE STUDIES

When catalase activity is negligible in a tissue/species, inhibition of the GSH‐ and Trx‐dependent pathways for the consumption of H_2_O_2_ allows to measure rates of superoxide/H_2_O_2_ formation while minimizing bias and/or imprecision. As explained above, this approach eliminates potential interspecific biases and increases sensitivity, and thereby provides novel opportunities to work with substrate/effectors combinations that better recapitulate in vivo rates and sources of superoxide/H_2_O_2_.

One limitation of using CDNB and auranofin, however, is potential species‐specific off‐target effects (Treberg et al., 2018). For example, in our recent study (Munro et al., 2019), the protocols developed for rat skeletal muscle mitochondria required adaptation to work with murine mitochondria, which generally seem more fragile (Treberg et al., 2018). Specifically, the concentration of auranofin was reduced from 2 to 0.5 µM, and the duration of the CDNB pretreatment was reduced from five to four minutes reaction time at room temperature in order to respect our threshold of a 5% maximal effect on coupled respiration in convergent electron flow (glutamate + malate + succinate + ADP). Hence, for each species/tissue contemplated, both the CDNB pretreatment and the use of auranofin should be tested for at least a number of parameters of mitochondrial energetics linked to the formation of H_2_O_2_ (Munro et al., 2016; Treberg et al., 2018). Moreover, direct addition of CDNB to the reaction milieu is known to cause a large increase in the rate of superoxide/H_2_O_2_ formation as a side effect (Liu et al., 2002). Therefore, inhibiting matrix consumers of H_2_O_2_ requires a CDNB pretreatment that eliminates unreacted CDNB. These time‐consuming additional centrifugation steps typically cause a further 50% loss in mitochondrial yield.

It might be worth asking whether or not measurements of consumption are more important than measurements of production for addressing the mitochondrial oxidative theory of aging. Mitochondria generally have much higher capacities for the consumption of H_2_O_2_ than for its production, which is reflected by the fact that over 50% of H_2_O_2_ production is lost to matrix consumers and masked from traditional H_2_O_2_ efflux assays. In addition, consumers of H_2_O_2_ in the matrix act as defenses not only against endogenous H_2_O_2_, but also against H_2_O_2_ of cytosolic origin, which may be important for some cell types and/or under certain physiological conditions (Brown & Borutaite, 2012). To illustrate this principle, the knockdown of the mitochondrial‐specific thioredoxin reductase (TRx2) not only affects matrix [H_2_O_2_], but also that of the cytosol during tert‐butyl hydroperoxide challenges of H9c2 cardiac myoblasts (Dey et al., 2016). Furthermore, site‐specific inhibitors of mitochondrial ROS formation have been found for the major ROS producing sites, which do not affect mitochondrial energetics (see Orr et al., 2013; Orr et al., 2015); however, to our knowledge, they have not yet been shown to produce life extension. In contrast, at least two antioxidants targeted to mitochondria (catalase and the SQk1 ions) proved capable of extending lifespan/healthspan of murine cohorts (Schriner et al., 2005; Shabalina et al., 2017; Skulachev et al., 2009). In this regard, measuring the integrated capacity of mitochondria to consume H_2_O_2_ is much simpler to conduct and less subject to caveats than measuring superoxide/H_2_O_2_ formation rate (Munro et al., 2016).

## 11 FURTHER IMPROVING COMPARATIVE STUDIES 1) ELIMINATING MITOCHONDRIAL DENSITY MARKERS

Previous comparative studies have almost exclusively standardized mitochondrial H_2_O_2_ efflux to mg of mitochondrial protein. The use of this marker of mitochondrial density has been questioned for the purpose of interspecific comparisons as it poorly associates with respiratory capacity relative to other commonly used markers (Hulbert, Turner, Hinde, Else, & Guderley, 2006). Perhaps more concerning is the possibility that relying on mg of mitochondrial protein could produce a negative relationship between longevity and the rate of mitochondrial superoxide/H_2_O_2_ formation as an artifact. Indeed, there is direct evidence of a negative relationship between mitochondrial density and longevity in mammalian liver (discussed in Passos, von Zglinicki, & Kirkwood, 2007), although the data for that relationship involve species for which body mass increases along with longevity (except for human), which could partly explain the lower mitochondrial density in long‐lived species (Hoppeler et al., 1984). Nevertheless, a significant negative relationship exists between mass‐specific basal metabolic rate and lifespan across 267 mammalian species (Hulbert et al., 2007). When put in perspective, with the strong relationship between parameters of metabolic rate such as *V*_O2max_, or basal metabolic rate, and the density of mitochondria within cells (Guderley, Turner, Else, & Hulbert, 2005; Weibel & Hoppeler, 2005), the negative relationship between longevity and mitochondrial density appears more than plausible. Because mitochondrial pellets recovered after differential centrifugation are never pure, a lesser mitochondrial cell density should result in lower ETS enzyme content (ROS producing enzymes) per mg of total protein in the mitochondrial pellet. In turn, this could result in the false determination of a negative relationship between longevity and mitochondrial ROS formation rate when resorting to mg of total protein in the mitochondrial isolate as a marker. Indeed, the mitochondrial density within a cell can vary by up to a factor of three to four between species, as can cristae density (Weibel, Bacigalupe, Schmitt, & Hoppeler, 2004).

The use of Percoll gradients to resolve the concern of pellet impurity is both time‐consuming and expensive, and has never been done in previous comparative studies of longevity addressing mitochondrial ROS. In addition, the mitochondrial density of ROS producing enzymes remains highly variable across species and strongly associated with metabolic rate (Guderley et al., 2005). Therefore, an inverse association between longevity and H_2_O_2_ efflux should be expected simply because of a lowed density of ROS producing enzymes relative to total mitochondrial protein in long‐lived species, even if impurities are eliminated from the mitochondrial isolate. In this scenario, the data generated will not allow conclusions to be drawn regarding whether or not mitochondria of long‐lived species produce less superoxide/H_2_O_2_ per unit of ETS complexes, nor whether or not they produce less superoxide/H_2_O_2_ relative to the capacity of matrix antioxidants. Standardizing mitochondrial superoxide/H_2_O_2_ formation rate to a marker of the TCA cycle, or an enzyme of the ETS, should provide more meaningful data. However, a systematic change in the ratio of one of these enzymes relative to those directly implicated in the production of ROS may well underlie a lower ROS formation rate in long‐lived species (metabolic re‐routing). Ultimately, all markers of mitochondrial density are potentially problematic (Hulbert et al., 2006; Larsen et al., 2012), as their content or activity may be potentially associated with longevity in one way or the other, creating another bias in the relationship.

Addressing the recent vision of the mitochondrial oxidative theory of aging requires consideration of the rate of ROS formation *along with* the rate of elimination. Fortunately, there is a simple and elegant solution to this problem: dividing true mitochondrial H_2_O_2_ formation rate (after inhibition of matrix antioxidants), for any given substrate condition, by the integrated measure of maximal rate of elimination (with malate, as discussed above). The primary variables (rate of production and elimination) are free of bias if measured as suggested herein, and dividing one by the other eliminates any bias related to the use of a marker of mitochondrial density. As a result, this unitless *oxidant index* is a simple, meaningful proxy of the degree of oxidative insult sustained by matrix macromolecules, which cuts right to the question asked by the theory—that is, mitochondria of long‐lived species should alleviate self‐inflicted (matrix) oxidative damages. We therefore suggest this oxidant index is the best means of comparing the level of chronic mitochondrial oxidative burden as part of evaluating the mitochondrial oxidative theory of aging (with the acknowledgment that repair mechanisms are also important).

## 12 FURTHER IMPROVING COMPARATIVE STUDIES 2) ASSESSING MnSOD

Past studies likely focussed on estimating rates of H_2_O_2_ efflux alone based on the premise that most oxidative damage is inflicted after Fenton reactions, which result in the formation of hydroxyl radicals from H_2_O_2_. Nevertheless, a non‐negligible contribution to the oxidative insult inflicted upon matrix components is directly due to superoxide, before its dismutation to H_2_O_2_. This component of the oxidative burden is left unaccounted for by the measures used in past studies, as well as those for (true) rates of H_2_O_2_ formation and elimination that we suggest herein. Simply measuring the specific activity of MnSOD would suffice to fill this knowledge gap. Superoxide cannot cross membranes; hence, its matrix concentration depends solely on its rate of formation and dismutation. In other words, at a constant rate of formation, lower MnSOD activity will result in higher superoxide levels as the level of superoxide will rise until its rate of dismutation once again matches the rate of production. The activity of the MnSOD is not dependent on other aspects of mitochondrial bioenergetics and can thus be simply measured with commercial kits on frozen biological fractions after chloroform/methanol extraction to separate it from that of the Cu/ZnSOD (Andziak et al., 2005).

## 13 THE NMR VERSUS THE MOUSE: A TEST CASE OF A NOVEL APPROACH

We recently reassessed the influential comparison between the long‐lived NMR and the mouse bearing in mind the pitfalls and possibilities described above (Munro et al., 2019). Mitochondria were isolated from skeletal muscle and heart, and we measured rates of H_2_O_2_ formation (after inhibition of matrix consumers of H_2_O_2_) and H_2_O_2_ clearance capacities.

The first striking finding of our study was that for skeletal muscle mitochondria three different conclusions emerged concerning the rate of H_2_O_2_ formation, depending on the marker of mitochondrial density considered. When standardizing to mg of mitochondrial proteins, citrate synthase, and oxygen consumption capacities, the global trend across multiple substrates conditions was for a lesser, equal, and higher rate of H_2_O_2_ formation by mitochondria from NMRs, respectively. This confusion is problematic if we are to reach a consensus about the implications of ROS in longevity—that is, all three of these markers have been used and considered the best at least once in past studies. This clearly highlights the issues described above regarding the use of traditional markers of mitochondrial density.

The second major finding was that the H_2_O_2_ clearance capacity of NMR mitochondria was strikingly greater than that of mouse mitochondria for both tissues across all substrate conditions as well as across all markers of mitochondrial density used to standardize data. This is a novel finding of clear interest to the comparative study of longevity. The fact that this pair of species clearly differs in their consumption capacity but not in their absolute rate of H_2_O_2_ formation illustrates the possibility that any negative relationship between longevity and mitochondrial H_2_O_2_ efflux, found in previous comparative studies, could in fact be explained by greater matrix antioxidant capacity in the long‐lived species.

A third major finding was that NMR skeletal muscle and heart mitochondria rely more so on the GSH‐dependent pathway for the consumption of H_2_O_2_, whereas the mouse relies more on the Trx‐dependent one. This finding illustrates how measuring the activity or expression of only one of the peroxidases (e.g., the GSH‐peroxidase) would have led to an incorrect conclusion about differences between the two species with regard to their matrix H_2_O_2_ detoxifying capacities, as discussed above.

Interestingly, an in‐depth look at figures 2 and 3 in Munro et al. (2019) shows that addition of ADP tended to decrease H_2_O_2_ formation more so for the NMR than for the mouse. Addition of ADP will increase the relative contribution of site I_F_ to the overall superoxide/H_2_O_2_ formation rate. As suggested above, if a downregulation of superoxide/H_2_O_2_ is part of the suite of adaptations driven by evolution to increase lifespan, then it may be expected to act primarily at site I_F_, which is relatively important in physiological conditions.

A fourth major finding further illustrates that it is possible to confound higher matrix antioxidant capacity with lower superoxide/H_2_O_2_ formation rates. We recently acquired H_2_O_2_ efflux data on heart mitochondria of NMRs and mice (same strain and same procedure, except for not inhibiting matrix antioxidants) in the context of a study on the tolerance to hypoxia (manuscript in preparation). In this more recent work, we found significantly higher rates of H_2_O_2_ efflux for the mice in the three conditions of succinate, succinate + rotenone, and succinate + rotenone + ADP. Similarly, Lambert et al. (2007) found a trend in the same direction for the same species/tissue and for the same general experimental conditions. Had they increased the sample size, and they might have come to the same significant result that the mouse mitochondria produce more than those of the NMR. In contrast, after inhibiting matrix antioxidants (Munro et al., 2019), we found that NMR mitochondria had higher absolute values for these conditions, even though the contrasts were not significant. Mitochondria with much greater activity of matrix consumers of H_2_O_2_ can therefore present significantly lower efflux (measured using traditional methods), even though (true) rates of superoxide/H_2_O_2_ formation are the same.

We did not measure the specific activity of MnSOD in our recent primary contribution partly because it had already been found to be significantly greater in liver of young and intermediately aged NMRs as compared to mice (Andziak et al., 2005). Finding the same difference for heart and skeletal muscle in our primary contribution would have been a worthwhile contribution to fully demonstrate greater activity for the entire suite of matrix antioxidants in the long‐lived species.

Finally, results of our re‐assessment of the NMR versus mouse comparison unequivocally support the more recent mitochondrial oxidative hypothesis of aging—that is, the oxidant index was lower for the NMR for both tissues in all conditions of substrates tested. This contrasts with conclusions of a previous study comparing heart mitochondria H_2_O_2_ efflux for the same two species, which concluded to the absence of a difference (Lambert et al., 2007).

## 14 EVOLUTIONARY PERSPECTIVE POSSIBLY UNDERLYING A MODULATION OF MATRIX ANTIOXIDANTS

The finding that the NMR does not differ from the mouse for its rate of superoxide/H_2_O_2_ formation, but rather in its augmented matrix antioxidant capacities might come as a little shift of paradigm, but is this finding really surprising? It had been suggested that reducing ROS formation at the source should be energetically more efficient than intercepting them; an influential idea that may have directed past studies at focussing primarily on rates of ROS formation (Barja, Cadenas, Rojas, Lopeztorres, et al., 1994). However, we now know that mitochondrial ROS play a key role in cellular signaling (Mailloux & Treberg, 2016; Pamenter, 2014), which can be site‐specific (Scialo et al., 2017). Furthermore, it has seldom been considered that modulating the capacity of the matrix antioxidants should be simpler, from an evolutionary point of view, than decreasing the initial rate of superoxide/H_2_O_2_ formation at various production sites. This is because matrix antioxidants only involve a limited number of nDNA encoded genes. In contrast, the sites of ROS formation are found at the core catalytic subunits of the ETS complexes, which are co‐encoded by mtDNA and nDNA, thus requiring co‐evolution of the two genomes (Bar‐Yaacov, Blumberg, & Mishmar, 2012).

## 15 CONCLUSION

The capacity of mitochondria to produce or consume ROS can only be investigated in isolated mitochondria because the cellular environment also contributes to the production and consumption of H_2_O_2_, a ROS species that can diffuse across biological membranes (Bienert & Chaumont, 2014). Adding to this concern, the notion according to which mitochondria are net sources of ROS in the cell might have been adopted prematurely; there may be little evidence to support this contention (Brown & Borutaite, 2012). In line with this, recent mechanistic developments in our understanding of mitochondrial ROS metabolism support a role for mitochondria as regulators of H_2_O_2_ in the cell (Munro et al., 2016; Munro & Treberg, 2017; Starkov et al., 2014; Treberg et al., 2015). This changing contextual framework does not exclude a role for the oxidation of matrix biomolecules such as mtDNA as one of the drivers of senescence; however, it demands a change in the theoretical and methodological approaches employed to answer the question. The methods suggested herein have been designed to circumvent caveats of past studies while focussing more carefully on the latest theoretical developments regarding the involvement of oxidative processes in aging. These methods may unravel novel correlates of longevity, pertaining to mitochondrial ROS metabolism, and hopefully untie apparent conundrums in this debated field of research.

## CONFLICT OF INTEREST

None declared.
